# Contactless Respiratory Waveform Estimation Using a Depth Camera and AI-Based Body Detection

**DOI:** 10.3390/s26113476

**Published:** 2026-06-01

**Authors:** Yuto Kojima, Toru Higaki, Hirotaka Inoue, Bisser Raytchev, Yanlei Gu, Yuko Nakamura

**Affiliations:** 1 Graduate School of Advanced Science and Engineering, Hiroshima University, 1-4-1 Kagamiyama, Higashi-Hiroshima City 739-8527, Japan; bisser@hiroshima-u.ac.jp (B.R.); guyanlei@hiroshima-u.ac.jp (Y.G.); 2 Department of Electrical Engineering and Information Science, National Institute of Technology (KOSEN), Kure College, 2-2-11 Agaminami, Kure City 737-8506, Japan; hiro@kure-nct.ac.jp; 3 Department of Diagnostic Radiology, Kasumi Campus, Hiroshima University, 1-2-3 Kasumi, Minami-ku, Hiroshima City 734-8551, Japan; yukon@hiroshima-u.ac.jp

**Keywords:** computed tomography, contactless respiratory monitoring, respiratory waveform estimation, depth camera, AI-based body detection, patient monitoring

## Abstract

Computed tomography (CT) examinations pose challenges for continuous patient observation, particularly when adverse events such as severe reactions to contrast media occur. To improve patient monitoring in such situations, this preliminary study proposes a contactless method for respiratory waveform estimation using a depth camera and AI-based body detection. The method identifies anatomically relevant respiratory regions and extracts depth-based motion signals while subjects are seated facing the camera, which is positioned approximately 2 m away. Performance was evaluated experimentally using a wearable force-sensor respiration belt as the reference. Quantitative assessment was conducted using waveform error metrics, Pearson correlation coefficients, respiratory-rate agreement, and Bland–Altman analysis, while qualitative analysis was used to examine the influence of clothing conditions on measurement performance. The results show that the proposed method can provide stable respiratory waveform estimation, with the chest region yielding the lowest waveform error and the highest correlation among the evaluated ROIs. Bland–Altman analysis further indicated small systematic errors in respiratory-rate estimation, although variability-related indices were affected by ROI selection and clothing conditions. These findings support the feasibility of the proposed approach for contactless respiratory monitoring during CT examinations and indicate that the main contribution of this study is to clarify the importance of anatomical ROI selection for robust waveform extraction under CT-oriented monitoring conditions.

## 1. Introduction

Computed tomography (CT) is widely used in routine clinical practice, and contrast-enhanced CT plays an important role in improving diagnostic performance. Although modern contrast media are generally safe, acute adverse reactions still occur and, in rare cases, may progress to severe and life-threatening conditions. In such situations, abnormal respiratory changes can be among the earliest observable signs of patient deterioration. In particular, rapid breathing patterns, such as tachypnea or hyperventilation, may indicate acute distress and, therefore, require careful attention during contrast-enhanced examinations.

In CT examinations, however, continuous patient observation is not always straightforward. Because the operator and patient are physically separated during image acquisition, subtle physiological changes may be missed until they become more apparent. This practical limitation motivates the need for a monitoring approach that can continuously assess respiratory status without requiring additional physical contact with the patient. A contactless system would be especially valuable in CT-related settings, where patient comfort, workflow simplicity, and rapid recognition of physiological change are all important.

Among possible contactless sensing modalities, depth cameras are attractive because they can capture subtle body-surface motion without attaching devices to the subject. Nevertheless, previous studies have largely focused on whether respiratory waveforms can be estimated from depth information and how closely the estimated waveform matches a reference waveform. For translation into clinical use, accurate and stable estimation of the respiratory waveform itself is especially important, because waveform fidelity underpins subsequent interpretation of respiratory timing, rate, and pattern changes. Practical respiratory monitoring should therefore be evaluated not only in terms of signal agreement but also in terms of robustness under realistic conditions and feasibility for continuous observation.

The present paper is an extended version of our conference paper, “Breath Waveform Measurement System Using Contact-less Devices,” presented at IDAACS 2025 [1]. Compared with that earlier report, the present journal manuscript provides a more detailed description of the depth-camera and AI-based body-detection framework and a broader evaluation of respiratory waveform estimation performance in a CT-related monitoring context.

In this study, we investigate a depth-camera-based contactless respiratory monitoring method, with particular emphasis on accurate respiratory waveform estimation. First, we expand the evaluation framework by introducing multiple complementary assessment metrics rather than relying on a limited technical comparison. Second, we examine the method from the perspective of clinical practicality, focusing on whether stable waveform acquisition can be achieved in CT-related environments. Third, we assess whether the estimated waveform preserves basic cycle information by evaluating respiratory-rate agreement with the reference waveform. From this perspective, the present work positions contactless respiratory measurement primarily as a waveform-estimation problem and as a foundational step toward broader respiratory monitoring.

## 2. Related Work

Respiratory monitoring has been studied using both contact-based and contactless sensing approaches. Contact-based devices, such as respiratory belts and other wearable sensors, can provide direct physiological measurements and are often used as reference waveforms in experimental studies. However, these methods require sensor attachment, increase preparation time, and may reduce comfort or flexibility in routine examinations. For this reason, contactless approaches have attracted increasing interest as a means of monitoring respiration while minimizing patient burden and preserving ordinary clinical workflow [2,3,4].

Among contactless technologies, depth-camera-based methods are particularly relevant because they can capture subtle body-surface motion associated with breathing [2,5]. Previous studies have shown that respiratory waveforms and respiratory rate can be estimated from depth changes around the torso, chest, or abdomen. At the same time, these studies have indicated that estimation accuracy depends strongly on region-of-interest (ROI) selection, signal extraction strategy, subject posture, and environmental noise [5,6]. In many cases, the main emphasis has been placed on waveform similarity to a reference waveform or on average respiratory-rate estimation under comparatively controlled conditions [7]. For example, Silverstein and Snyder [7] presented a markerless Kinect v2 approach for respiratory motion tracking in four-dimensional CT and radiotherapy gating, focusing on user-selected depth points and agreement with existing gating systems. Prochazka et al. [5] demonstrated that Kinect image, depth, and infrared sensors can capture breathing-related signals from selected ROIs, highlighting the importance of ROI design and signal processing for physiological interpretation. Addison et al. [8] further reported continuous non-contact monitoring of respiratory rate and tidal volume using a depth-sensing camera, indicating that waveform extraction can support not only rate estimation but also volume-related respiratory surrogates. Imano et al. [9] also showed that depth-camera measurements from the chest and abdominal regions can be used to estimate tidal volume and respiratory rate in older participants, again emphasizing the importance of region selection for practical monitoring. More recently, Addison et al. [6] and MacLeod et al. [4] reported robust depth-camera-based respiratory-rate monitoring under broader and more challenging conditions, including variations in posture, body coverings, patient motion, and anesthesia-related disturbances. In parallel, Chavernac et al. [10] extended Azure Kinect-based respiratory sensing toward real-time current-volume estimation in pediatric intensive care, suggesting the broader clinical relevance of depth-based thoracoabdominal measurement beyond simple respiratory-rate estimation.

Despite these advances, several issues remain before such methods can be considered clinically practical. First, broader evaluation frameworks are needed to assess performance from multiple complementary viewpoints rather than from a single error measure. Second, practical usability in CT-related environments remains important because continuous monitoring must remain stable under realistic conditions rather than only in technically ideal settings [7]. Third, relatively less attention has been paid to whether estimated respiratory waveforms remain sufficiently stable and interpretable to support downstream respiratory assessment under practical measurement conditions. These gaps motivate the present study, which examines depth-camera-based respiratory monitoring primarily as a clinically relevant waveform-estimation task.

## 3. Materials and Methods

### 3.1. Overview

This study evaluated a contactless respiratory measurement framework based on depth imaging and AI-based body detection. The overall pipeline consisted of synchronized acquisition of depth data and a reference waveform, body detection and torso localization, selection of respiratory ROIs, generation of a depth-derived respiratory waveform, and comparison of the estimated waveform with the reference waveform. Measurements were performed in a seated, front-facing posture to minimize gross body motion and to examine the respiratory component under CT-related monitoring conditions [4].

**Figure 1:**
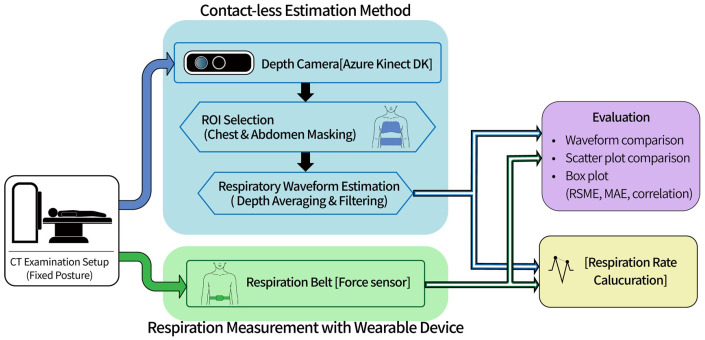
Overall measurement framework for synchronized acquisition of depth-camera data and signals from the wearable force-sensor respiration belt.

Figure 1 illustrates the overall measurement framework, in which a depth camera and a wearable force-sensor respiration belt were used simultaneously to acquire respiratory information.

### 3.2. Reference Respiratory Sensor

To obtain a reference waveform, a wearable force-sensor respiration belt (NaRiKa, Tokyo, Japan)was used during each measurement. The belt directly captured physical expansion and contraction associated with breathing and served as the reference for evaluation. To ensure a fair comparison between contact-based and contactless sensing, the raw belt waveform was analyzed instead of relying on the device’s internal respiratory-rate output. Respiratory rate was then derived from the belt waveform using the same peak-based procedure applied to the depth-derived waveform, so that differences in performance could be attributed to the sensing modality rather than to different post-processing algorithms [4].

### 3.3. Depth Camera Acquisition

Contactless measurements were obtained using an depth camera (Azure Kinect DK, Microsoft, WA, USA). The software and SDK versions used for depth-camera acquisition, body tracking, reference-sensor acquisition, and subsequent analysis are summarized in Table 1.

The camera was positioned approximately 2 m in front of the seated subject so that the upper body could be continuously observed throughout each recording. To capture subtle respiratory motion while maintaining practical computational cost, the depth sensor was operated in wide field-of-view 2 × 2 binned mode, with a spatial resolution of 512 × 512 pixels and a frame rate of 30 frames/s. These settings were selected to provide sufficient temporal resolution for respiratory monitoring while preserving stable depth acquisition over the thoracoabdominal region [10,11].

### 3.4. AI-Based Body Detection and ROI Selection

To suppress background influence and restrict the analysis to anatomically relevant regions, AI-based body tracking was applied to each frame using the Microsoft Azure Kinect Body Tracking SDK, a skeletal tracking framework provided by Microsoft for the Azure Kinect DK. The SDK estimates body joints by combining two-dimensional joint detection from infrared images using a convolutional neural network with depth-map-based model fitting, thereby enabling three-dimensional skeletal tracking rather than image-plane body localization alone [12,13]. In the present study, body landmarks around the neck, pelvis, and shoulders were used to define a torso-centered bounding region. A torso line connecting the neck and pelvis was then used as the main vertical reference for ROI definition. To reduce artifacts from the arms and surrounding background, horizontal masking was applied according to the detected shoulder width [5].

Let $I_{t}$ and $D_{t}$ denote the infrared image and depth map acquired at frame *t*, respectively. The body tracking system estimated a set of skeletal landmarks,

(1) $$J_{t}={\left\{p_{j,t}\right\}}_{j=1}^{J}$$

where $p_{j,t}$ represents the image-plane position, or the corresponding three-dimensional position when available, of joint *j* at frame *t*. Let $p_{N,t}$ and $p_{P,t}$ denote the detected neck and pelvis landmarks, respectively. The torso axis was defined as

(2) $$a_{t}=p_{P,t}-p_{N,t},$$

and a normalized coordinate along the torso was introduced as

(3) $$s_{t}\left(x\right)=\frac{{\left(x-p_{N,t}\right)}^{⊤}a_{t}}{∥a_{t}{∥}^{2}},\;0\leq s_{t}\left(x\right)\leq 1,$$

where $x={\left(u,v\right)}^{⊤}$ is a pixel coordinate. The value $s_{t}=0$ corresponds approximately to the neck position, whereas $s_{t}=1$ corresponds approximately to the pelvis position. Using the shoulder width $w_{t}$ estimated from the left and right shoulder landmarks, the torso mask was represented as

(4) $$M_{t}=\{x:0\leq s_{t}\left(x\right)\leq 1,\;|r_{t}\left(x\right)\left|\leq \alpha w_{t}\right\},$$

where $r_{t}\left(x\right)$ is the horizontal coordinate relative to the torso axis and α is a scaling parameter used to exclude the arms and background.

Within the isolated torso region, the body area was divided into five segments along the torso line. For ROI *R*, the corresponding pixel set was defined as

(5) $$M_{R,t}=\left\{x\in M_{t}:s_{R}^{\min}\leq s_{t}\left(x\right)<s_{R}^{\max}\right\}.$$

The second and fourth segments from the top were selected as representative chest and abdominal ROIs, respectively, while the broader torso region was retained as a comparison region. This strategy enabled direct comparison of respiratory-estimation performance across different anatomical areas and emphasized regions in which cyclic surface displacement was expected to be strongest.

### 3.5. Respiratory Waveform and Rate Estimation

For each frame, the average depth value inside the selected ROI was calculated to obtain a raw time-series signal corresponding to respiratory surface motion. If $V_{R,t}\subseteq M_{R,t}$ denotes the subset of valid depth pixels in ROI *R*, the raw depth-derived waveform was calculated as

(6) $$z_{R}\left(t\right)=\frac{1}{|V_{R,t}|}\sum_{x\in V_{R,t}}D_{t}\left(x\right).$$

This averaged depth signal represents cyclic displacement of the body surface caused by respiration. Temporal preprocessing was then applied to suppress measurement noise and baseline drift while preserving the dominant breathing component. In the present implementation, smoothing was used to reduce high-frequency fluctuations before respiratory peaks were detected:

(7) $${\tilde{z}}_{R}\left(t\right)=S\left\{z_{R}\left(t\right)\right\},$$

where S{·} denotes the smoothing operation.

For waveform comparison across sensing modalities, both the depth-derived waveform and the wearable-belt waveform were normalized to the range [0, 1] over short windows of several seconds. For a temporal window $W_{k}$, the normalized depth-derived waveform and reference waveform were defined as

(8) $${\hat{z}}_{R}\left(t\right)=\frac{{\tilde{z}}_{R}\left(t\right)-\min_{\forall \tau \in W_{k}}{\tilde{z}}_{R}\left(\tau \right)}{\max_{\tau \in W_{k}}{\tilde{z}}_{R}\left(\tau \right)-\min_{\forall \tau \in W_{k}}{\tilde{z}}_{R}\left(\tau \right)},\;t\in W_{k},$$

and

(9) $$\hat{b}\left(t\right)=\frac{b\left(t\right)-\min_{\forall \tau \in W_{k}}b\left(\tau \right)}{\max_{\forall \tau \in W_{k}}b\left(\tau \right)-\min_{\forall \tau \in W_{k}}b\left(\tau \right)},\;t\in W_{k},$$

respectively, where b(t) is the reference belt waveform. This windowed normalization reduced the influence of sensor-specific units and slow positional offsets, allowing subsequent error metrics to emphasize local waveform agreement.

Respiratory rate was estimated from the smoothed waveform by identifying local maxima corresponding to repeated phases of respiratory expansion. To avoid false detections caused by short-term fluctuations, peaks occurring within 0.3 s of the preceding peak were excluded from the final count. Let ${\left\{t_{m}\right\}}_{m=1}^{M}$ be the retained peak times in the smoothed depth-derived waveform. The respiratory period was estimated from successive peak intervals as

(10) $$\Delta t_{m}=t_{m+1}-t_{m},$$

and the respiratory rate in breaths per minute was calculated as

(11) $$RR_{R}\left(t\right)=\frac{60}{{\mathrm{median}}_{m}\left(\Delta t_{m}\right)}.$$

The same procedure was applied to the wearable-belt waveform so that differences in respiratory-rate estimates reflected the sensing modality rather than differences in post-processing algorithms.

### 3.6. Experimental Protocol and Evaluation

Experiments were conducted with four participants. Each participant was asked to maintain a seated, front-facing posture and to breathe naturally without explicit control of respiratory depth or rhythm. Each recording session lasted slightly more than one minute, and depth-camera data were acquired simultaneously with data from the wearable force-sensor respiration belt. Because clothing can influence the visible surface displacement captured by the depth camera, clothing conditions were also recorded for each participant, as summarized in Table 2.

For the waveform-level evaluation, a continuous segment of each recorded signal was used for qualitative inspection of temporal correspondence between the estimated and reference waveforms. Quantitative assessment was performed using root mean square error (RMSE), mean absolute error (MAE), and the Pearson correlation coefficient computed from the waveforms after windowed normalization. For *T* samples, these metrics were defined as

(12) $$\begin{array}{cc} {\mathrm{RMSE}}_{R} & =\sqrt{\frac{1}{T}\sum_{t=1}^{T}{\left({\hat{z}}_{R}\left(t\right)-\hat{b}\left(t\right)\right)}^{2}}, \end{array}$$

(13) $$\begin{array}{cc} {\mathrm{MAE}}_{R} & =\frac{1}{T}\sum_{t=1}^{T}\left|{\hat{z}}_{R}\left(t\right)-\hat{b}\left(t\right)\right|, \end{array}$$

and

(14) $$\rho_{R}=\frac{\sum_{t=1}^{T}\left({\hat{z}}_{R}\left(t\right)-{\bar{z}}_{R}\right)\left(\hat{b}\left(t\right)-\bar{b}\right)}{\sqrt{\sum_{t=1}^{T}{\left({\hat{z}}_{R}\left(t\right)-{\bar{z}}_{R}\right)}^{2}}\sqrt{\sum_{t=1}^{T}{\left(\hat{b}\left(t\right)-\bar{b}\right)}^{2}}},$$

where ${\bar{z}}_{R}$ and $\bar{b}$ denote the temporal means of ${\hat{z}}_{R}\left(t\right)$ and $\hat{b}\left(t\right)$, respectively. In addition, respiratory-rate agreement between the depth-derived waveform and the reference waveform was examined as a supplementary measure of whether the estimated waveform preserved basic cycle information. Bland–Altman analysis was further used to assess systematic error and agreement in respiratory-rate estimates, with the bias defined as the estimated respiratory rate minus the force-sensor reference respiratory rate.

## 4. Results

### 4.1. Qualitative Analysis of Respiratory Waveforms and Correlation

**Figure 2:**
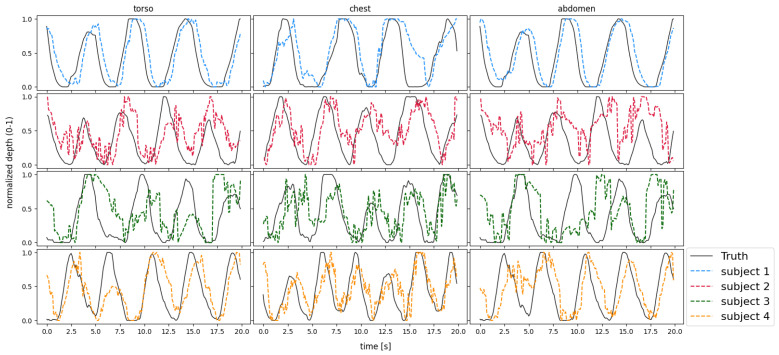
Comparison of estimated respiratory waveforms and reference waveforms across different regions of interest.

Figure 2 presents a qualitative comparison of the estimated respiratory waveforms with the reference waveform. Visual inspection of representative 20 s segments confirmed that the depth-based method successfully captured the fundamental respiratory rhythm. Although signal amplitude varied depending on the selected ROI, no clear phase shift was observed between the estimated and reference waveforms, indicating that the temporal structure of respiration was preserved.

Comparison among ROIs revealed clear differences in signal quality. The abdominal ROI often appeared to follow the reference waveform closely, whereas the chest ROI provided a similarly stable waveform with less sensitivity to local artifacts. In contrast, the whole-torso signal contained substantially more noise, suggesting that inclusion of non-respiratory regions degraded the respiratory estimate. Waveform quality also depended on clothing condition. In particular, Subjects 2 and 3, who wore thicker garments (Table 2), exhibited more pronounced high-frequency fluctuations than Subjects 1 and 4.

**Figure 3:**
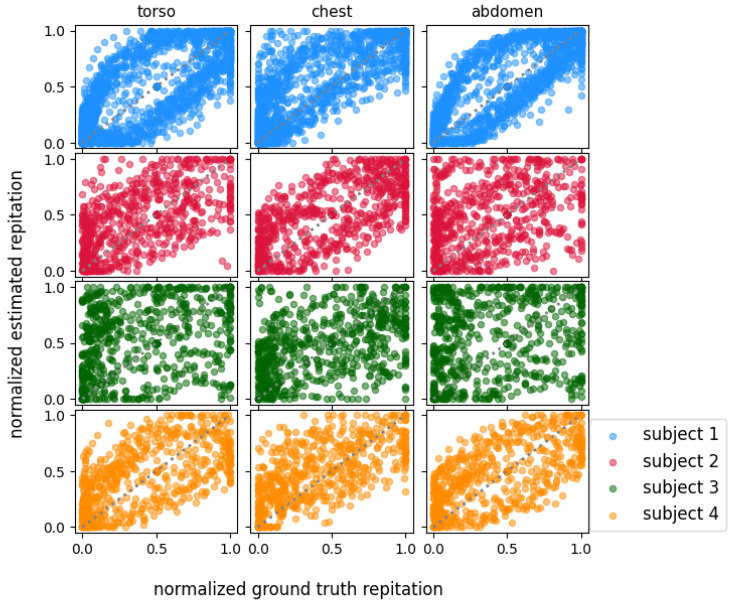
Scatter plots of normalized estimated depth values versus normalized reference values for each subject and region.

Figure 3 further illustrates the relationship between the normalized estimated depth values and the normalized reference values using scatter plots. These scatter plots were retained as a qualitative, sample-level visualization of waveform behavior across the entire analyzed signal, rather than as the primary method for assessing agreement in respiratory-rate estimation. A tighter concentration of points along the diagonal indicates better waveform agreement between the two measurements after normalization. Consistent with the waveform observations, the chest and abdominal ROIs showed stronger linear concentration than the whole-torso region. However, the scatter became broader in participants wearing thicker or looser clothing, suggesting that clothing thickness and folds interfered with the reliable capture of subtle thoracoabdominal surface motion.

### 4.2. Quantitative Error Evaluation and Impact of Clothing

**Figure 4:**
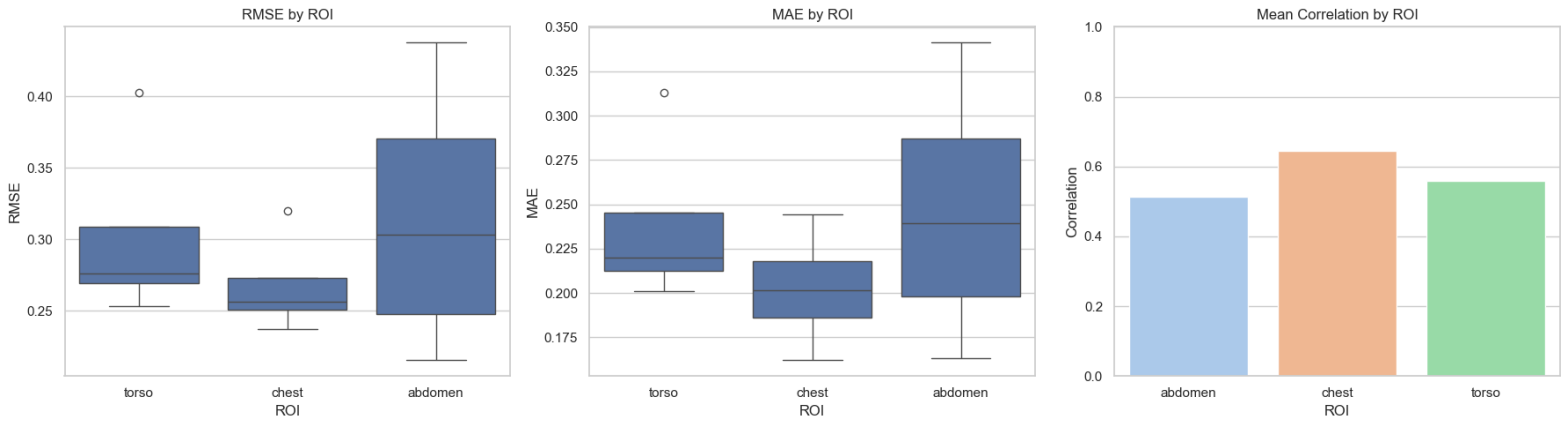
Quantitative performance comparison across different measurement regions, showing box plots for RMSE and MAE together with mean correlation coefficients.

Figure 4 summarizes the quantitative performance of each measurement region using RMSE, MAE, and the mean correlation coefficient. Although the abdominal ROI sometimes appeared favorable in the qualitative examples, evaluation over the entire dataset showed that the chest ROI provided the most consistent performance across subjects. Specifically, the chest ROI exhibited the lowest median RMSE and MAE values and the highest mean correlation with the reference waveform. In addition, the narrow interquartile range of the chest measurements indicated relatively stable estimation with limited dispersion.

By contrast, the abdominal ROI showed greater variability across participants. This instability was especially evident in subjects wearing thicker or looser clothing, as summarized in Table 2. Such clothing conditions likely attenuated or distorted the subtle surface displacement associated with abdominal respiration, thereby reducing the fidelity of depth-based measurements. Because chest motion is more directly associated with rib-cage expansion and tended to be less affected by folds and looseness of clothing in the present dataset, the chest ROI showed comparatively robust performance under the present measurement conditions.

Taken together, these results indicate that careful ROI selection is essential for stable respiratory waveform estimation. They also suggest that clothing is not merely a minor nuisance factor but a practical determinant of performance, especially when abdominal motion is used as the primary source of respiratory information.

### 4.3. Respiratory-Rate Agreement by Bland–Altman Analysis

**Figure 5:**
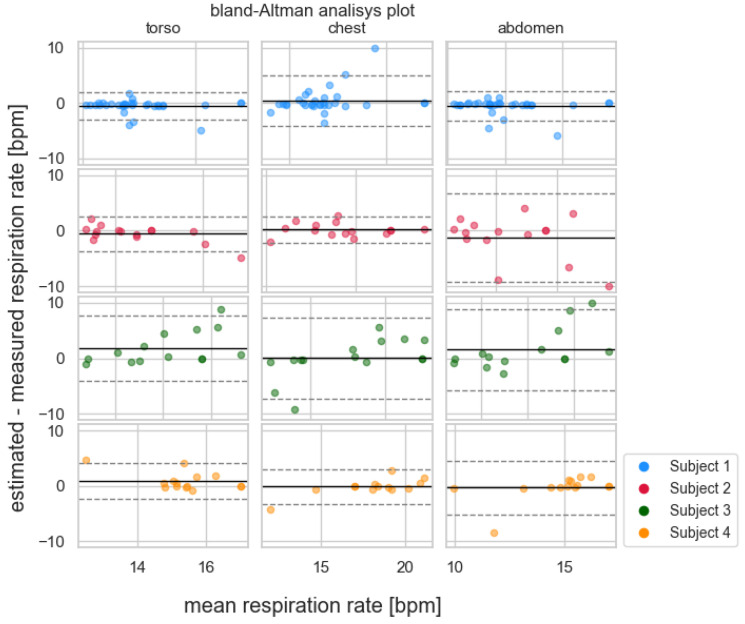
Bland–Altman plots comparing respiratory rates measured by the force sensor with respiratory rates estimated from depth-camera measurements across different ROIs.

Bland–Altman analysis was performed as the quantitative agreement analysis for respiratory-rate estimation, comparing the respiratory rate measured by the force sensor with the respiratory rate estimated from the depth-camera signals (Figure 5). Thus, whereas the scatter plots were used to qualitatively inspect waveform-level behavior over the analyzed samples, the Bland–Altman analysis was used to quantitatively evaluate respiratory-rate agreement. The corresponding bias, standard deviation (SD), and limits of agreement (LOA) are summarized in Table 3. With respect to bias, the anatomically restricted chest and abdominal ROIs showed smaller systematic errors than the whole-torso ROI. This finding was consistent with the qualitative evaluation, suggesting that restricting the measurement region to respiratory-relevant anatomical areas can reduce systematic deviation in the estimated respiratory rate.

In contrast, the SD and LOA-related indices were more favorable for the whole-torso ROI. This result was partly counterintuitive because restricting the ROI to regions with prominent respiratory motion was expected to improve the stability of respiratory-rate estimation. One possible explanation is that the local chest and abdominal ROIs were more strongly affected by clothing-related artifacts, including garment thickness, wrinkles, and looseness, which may have increased variability in the estimated respiratory rate. In addition, because this was a preliminary study with a limited sample size, subject-specific clothing conditions and individual variability may have had a relatively large influence on the Bland–Altman results. Therefore, the apparent advantage of the whole-torso ROI in variability-related indices should be interpreted with caution and validated in a larger cohort under more diverse clothing conditions.

## 5. Discussion

The present findings indicate that ROI selection is a critical determinant of performance in depth-camera-based respiratory monitoring. Although abdominal motion occasionally appeared visually similar to the reference waveform, quantitative analysis across the full dataset demonstrated that the chest ROI yielded the most stable and accurate waveform-level performance across participants. This result suggests that visual similarity alone does not provide a sufficient basis for judging practical measurement quality and further underscores the importance of evaluating respiratory estimation using multiple complementary metrics. More importantly, it shows that practical system design for CT-oriented monitoring should carefully consider differences among anatomically plausible torso regions rather than assuming equivalent performance.

The significance of this result becomes more apparent when considered in relation to prior studies. Silverstein and Snyder [7] reported that user-selected depth points obtained with Kinect v2 can provide respiratory traces suitable for radiotherapy gating; however, their framework was primarily directed toward consistency with established gating systems rather than systematic comparison of anatomically defined ROIs under practical clothing variation. Similarly, Prochazka et al. [5] demonstrated that depth and infrared sensing can recover breathing-related information from selected facial and thoracic regions, thereby supporting the general feasibility of region-based respiratory sensing; however, their study was broader in physiological scope and did not specifically address identification of the most robust torso ROI for stable waveform estimation in a CT-related setting. Accordingly, the present study extends these earlier reports by demonstrating that, even when multiple torso regions appear visually plausible, waveform-estimation performance differs systematically across ROIs under the tested conditions. In this respect, the contribution of the present work is not merely confirmatory; rather, it clarifies the practical importance of anatomical measurement-region selection for CT-related non-contact respiratory monitoring.

A second notable finding concerns the influence of clothing on measurement robustness. Thicker or looser garments increased noise and dispersion in the estimated signal, particularly in the abdominal ROI. This observation suggests that surface deformation associated with breathing may be partially obscured by clothing, thereby reducing the fidelity of depth-based measurements. By contrast, the chest region appeared less susceptible to such artifacts, presumably because rib-cage expansion generates more structurally consistent motion. From a practical perspective, this observation is relevant to clinical deployment, as patients in CT-related settings may wear garments of varying thickness and fit. The importance of robustness under realistic scene variation is consistent with the broader literature. Addison et al. [2] emphasized that depth-camera monitoring should be assessed beyond idealized technical agreement alone, and a subsequent study demonstrated strong respiratory-rate tracking under varied conditions [6]. MacLeod et al. [4] further showed that depth sensing can maintain respiratory-rate monitoring in an anesthesia environment despite patient motion, caregiver interaction, and body coverings. Relative to these studies, the present work places greater emphasis on waveform-level agreement and on the combined influence of ROI selection and clothing on the stability of the recovered respiratory signal. This combined analysis strengthens the methodological novelty of the study because it links measurement-region choice directly to clinically relevant robustness without reducing the design implication to a single universally preferred ROI.

Comparison with Chavernac et al. [10] is likewise informative. Their Azure Kinect-based system targeted real-time current-volume estimation in pediatric intensive care, thereby illustrating that depth sensing may support more comprehensive respiratory assessment in clinically demanding environments. Although the present study is more limited in scope, the results suggest that reliable waveform extraction from carefully selected and evaluated ROIs may provide an important foundation for such future extensions because inaccurate or unstable waveform recovery would directly constrain downstream estimation of respiratory rate, volume-related surrogates, or abnormal respiratory patterns. From this perspective, the present work represents an enabling step: before advanced respiratory indices can be estimated robustly, the measurement system must first account for how waveform-extraction stability depends on ROI selection.

Taken together, these findings support the feasibility of contactless respiratory monitoring in constrained examination environments. At the same time, the present study is limited by the relatively small number of participants and by evaluation under a restricted seated posture. Because this sample size is insufficient for the precise assessment of inter-subject variability, future supine clinical studies should include a larger number of participants to obtain more reliable performance estimates under conditions closer to actual CT examinations. In the next experimental phase with an increased sample size, blind source separation and motion decomposition will also be incorporated into the processing program, and their effectiveness in suppressing non-respiratory motion components, such as minor limb tremor and clothing deformation, will be quantitatively evaluated.

Another limitation is that the experiments were conducted during calm, natural breathing. Because abnormal respiratory patterns may occur in the context of adverse reactions to contrast media, future work should separately evaluate the responsiveness and tracking performance of the proposed method under simulated tachypnea, bradypnea, and breath-holding conditions.

## 6. Conclusions

This preliminary study evaluated a depth-camera-based contactless respiratory waveform-estimation method using AI-based body detection and anatomically defined ROIs. The results indicate that respiratory waveforms can be estimated without attaching sensors to the subject under the tested seated measurement conditions.

Among the evaluated ROIs, the chest region showed the most stable waveform-level performance, with lower waveform error and higher correlation than the abdominal and whole-torso regions. Bland–Altman analysis showed only small systematic errors in respiratory-rate estimation, although variability-related indices differed among ROIs. The results also suggest that clothing can affect depth-based waveform estimation, particularly when abdominal motion is used as the primary respiratory signal source.

These findings support the importance of anatomical ROI selection for robust contactless respiratory monitoring. However, because the present study was limited in sample size and measurement posture, further validation with more participants, supine CT-like positioning, and a broader range of respiratory conditions is required before clinical deployment.

## Figures and Tables

**Table 1:** Software and SDK versions used for data acquisition and analysis.

Software/SDK	Version	Purpose in This Study
Python	3.12.10	Signal processing and statistical analysis
Azure Kinect SDK	1.4.1	Depth and infrared acquisition
Azure Kinect Body Tracking SDK	1.1.2	Skeletal tracking and landmark detection
pykinect_azure	0.0.4	Python interface for Azure Kinect
godirect	1.2.1	Respiration-belt signal acquisition

**Table 2:** Clothing conditions recorded for each participant.

Subject	Clothing Description
Subject 1	Shirt
Subject 2	Thick hoodie
Subject 3	Thick sweater
Subject 4	T-shirt

**Table 3:** Bland–Altman statistics for respiratory-rate agreement between the force-sensor reference and depth-derived estimates. Bias was calculated as the estimated respiratory rate minus the force-sensor respiratory rate. All values are in breaths/min.

ROI	Bias	SD	LOA Lower	LOA Upper
Abdomen	−0.146	2.904	−5.839	5.546
Chest	0.141	2.201	−4.174	4.455
Whole torso	0.416	1.872	−3.253	4.084

## Data Availability

The data supporting the findings of this study are available from the corresponding authors upon reasonable request. The data are not publicly available because consent for the public disclosure of participant data was not obtained at the time of data collection.
